# An Attempt to Find a Suitable Biomass for Biochar-Based Polypropylene Biocomposites

**DOI:** 10.1007/s00267-018-1033-6

**Published:** 2018-03-28

**Authors:** Oisik Das, Nam Kyeun Kim, Mikael S. Hedenqvist, Richard J. T. Lin, Ajit K. Sarmah, Debes Bhattacharyya

**Affiliations:** 1 Department of Fibre and Polymer Technology- Polymeric Materials, School of Chemical Sciences and Engineering, KTH Royal Institute of Technology, Stockholm, 100 44 Sweden; 2 Centre for Advanced Composite Materials, Department of Mechanical Engineering, University of Auckland, Auckland, New Zealand; 3 Department of Civil and Environmental Engineering, Centre for Advanced Composite Materials, University of Auckland, Auckland, New Zealand

**Keywords:** Biomass, Biocomposite, Biochar, Wastes, Fire

## Abstract

Four biomass wastes (rice husk, coffee husk, coarse wool, and landfill wood) were added with biochar and polypropylene (PP) to manufacture biocomposites. Individual biomasses were tested for their combustion behavior using cone calorimeter. Biocomposites were analyzed for their fire/thermal, mechanical, and morphological properties. Wood had the most desirable comprehensive effect on both the mechanical and fire properties of composites. In particular, wood and biochar composite exhibited the highest values of tensile/flexural properties with a relatively low peak heat release rate. In general, application of waste derived biochar and biomasses drastically reduced the susceptibility of neat PP towards fire.

## Introduction

The application of organic wastes and residues in composite materials encourage an environmentally friendly and value-added path for resource conservation and recycling (Piri et al. 2018). Therefore, the potential for using waste derived biochar in the fabrication of composite materials has been recently explored to promote environmental waste management and sustainability. In particular, a loading amount of 24wt% biochar was found to be the most desirable for enhanced mechanical properties (Das et al. 2015a; Väisänen et al. 2017). Additionally, the surface area and carbon content of the biochar were deemed to be the most significant factors for developing composites with better performance properties (Das et al. 2015b). However, almost all the manufacturing and development of the biochar-based biocomposites included a single type of biomass as the co-reinforcement (Das et al. 2015a; Ayrilmis et al. 2015; Das et al. 2016; DeVallance et al. 2016). Hence, in order to impart versatility and effective waste management, it is imperative to study the suitability of different types of biomasses with biochar in a biocomposite for various performance requirements. Evidently, the same has also been pointed out in a recent work (Väisänen et al. 2016) about the necessity for incorporating several different types of biomasses in conjunction with biochar in biocomposites.

Keeping in mind the need for utilization of organic wastes, it is important that only common organic waste streams be used to manufacture biochar-based biocomposites. Organic wastes with the potential to degrade aided by bacteria could generate harmful greenhouse gases or leachates (Freed et al. (2004)). Therefore, for the purpose of reducing pollution, it is required to reuse the waste biomasses in manufacturing innovative materials. Several types of organic wastes, such as sunflower/corn stalks, rice husks, wool, waste paper sludge, etc., were already used to manufacture biocomposites in the past (Ashori and Nourbakhsh 2010; Bilal et al. 2013; Kim and Bhattacharyya 2016; Hamzeh et al. 2011). However, the synergistic behavior of these organic wastes and biochar in a composite is not yet fully understood. Some of the common and readily available organic wastes are: (1) rice husk owing to rice being a staple food in most parts of the World; (2) coffee husk from the global consumption of the beverage; (3) coarse wool as a result of large sheep population in New Zealand (NZ) which is often disposed of; and (4) landfill *wood* as a by-product of forestry waste in NZ (Zoccola et al. 2014). The addition of the aforementioned biomasses with biochar to fabricate biocomposites would simultaneously utilize organic wastes, create innovative materials for diverse applications and increase their values.

When biomass is heated to high temperatures (~>400–500 °C) in limited or complete absence of oxygen, the volatiles escape leaving behind a porous carbonaceous material, i.e., biochar (Gray et al. 2014). Manufacturing of biochar-based polymeric composites takes advantage of the porous surface structure allowing molten polymer to flow into them and consequently creating a mechanical interlocking between the matrix and the reinforcement. Moreover, the high surface area of biochar produced from high-temperature pyrolysis aids the dispersion of the particles in the polymer matrix (Ho et al. 2015). While the physical structure of biochar has been utilized for enhancing the mechanical properties of polymeric composites, its inherent carbonaceous nature, stable C-C covalent bonds amongst carbon molecules, and aromatic rings with high bond energy could be beneficial for imparting fire resistance to the composites. Composite materials have to pass stringent fire safety regulations in order to be employed in sectors, such as automotive, aviation, and building materials. The (relatively) high-temperature biochar with a high porosity was also found to resist combustion as the volatiles that could sustain burning already departed during its initial production (Liu et al. 2014). This fire resistant property of biochar could be extremely advantageous as both the polypropylene (PP) matrix (due to its hydrocarbon backbone), and the aforementioned biomasses are generally susceptible to burning.

All the past studies explored the effects of biochar and biomasses separately, and no investigation was conducted which studied the combined effects of biochar and biomasses in polymer based composites. This necessitates the need to comprehend the properties (mainly fire and mechanical) of composites containing both biochar and waste biomasses. From a sustainability perspective, the inclusion of both biochar and biomass, would allow the recycling of waste-based resources and the amount of synthetic polymer to be further reduced whereas, from the performance viewpoint, the dual reinforcements could result in a betterment of the composite’s properties. In addition, nowhere in the current literature, the reaction-to-fire properties of individual biomasses (without being integrated in a composite) are available. The data presented in this paper would aid in the comprehension whether a particular biomass would be beneficial for a composite’s fire properties along with its mechanical properties. Moreover, some of the biomass used in the current study, such as rice and coffee husks, are not widely studied in the composite field. Therefore, it is important to gain an insight into their effects (thermal, reaction-to-fire and mechanical) on composites to gauge their potentiality as reinforcements.

The central aim of this paper was to identify a waste biomass most suitable with biochar in a polypropylene (PP) based composite. Four different biomass wastes, namely rice husk (RH), coffee husk (CH), coarse wool (WL), and landfill wood (WD) were used to manufacture biochar (BC)/PP composites. The individual reaction-to-fire and thermal properties of all the biomasses along with the biochar were investigated. The resulting composites were also subjected to many tests in order to reveal their reaction-to-fire, thermal, mechanical, and morphological (both structural and post-combustion char) properties. The biomass exhibiting the best balance of mechanical and fire properties in biochar/PP biocomposites was identified in this investigation. Moreover, environmental and economic advantages of the selected biomass and biochar for the composites performance were also discussed by comparison with synthetic materials.

## Materials and Methods

### The Constituents

The biochar was prepared from landfill pine wood (WD) in an auger reactor at 900 °C for a residence time of 60 min at Taupo Carbon Producers, Taupo, NZ. The same landfill pine wood (feedstock for the biochar) was also used as one of the biomasses to be added into biochar/PP composites. The rice husk (RH) was obtained from a local rice milling plant in Pakistan whereas the coffee husk (CH) was collected from Segafredo Zanetti coffee factory in Auckland. Coarse wool (WL) fibers were provided by Bloch and Behrens Ltd, NZ, which was cleaned using non-ionic solutions in a scouring process to remove surface impurities. The moisture contents of the constituents were 1.5, 9.85, 9.79, 11.99 and 11.91% for biochar, rice husk, coffee husk, wool and wood, respectively. An electronic moisture analyzer (Model MA35, Sartorius, Germany) determined the moisture content of the constituents by detecting biomass weight loss after heating up to target temperature (100 °C) for 10 min. Polypropylene (PP) (Molpen HP 400 L) with a melt flow index (MFI) of 5.5 g/10 min was obtained from TCL HUNT. Clariant Ltd provided the maleic anhydride grafted polypropylene (MAPP) as a compatibilizer (Licocene PP MA 6452 Fine Grain TP).

### Biocomposite Manufacturing

The amounts of biomass (30 wt%), biochar (24 wt%), PP (42 wt%) and MAPP (4 wt%) used for all of the biocomposites manufactured in this study were based on results from a previous study (Das et al. 2015a) (Table 1). Before manufacturing, all the constituents (RH, CH, WL, WD, biochar and PP) were ground in a Retsch Mill (Model: SM 100; 230 V and 50 Hz) and passed through a 425 µm sieve. Biocomposite samples were produced through melt blending in a co-rotating Brabender® twin-screw extruder (Lab Tech Engineering Company Ltd. Scientific; Model: LHFS1–271822; S/N 020) followed by injection molding (Dr. Boy, GMBH 53577, Neustadt, Germany) to obtain the required sample configurations for the tests. The barrel temperature range in the extruder was 160–195 °C whereas the holding and the backpressure was set at 70 and 0.8 bar in the injection molding (same processing temperature as the extruder).

**Table 1:** Blend ratios of biomass composites

Samples	Biomass Type	Biomass (wt %)	Biochar (wt %)	PP (wt %)	MAPP (wt%)
RH+BC	Rice husk	30	24	42	4
CH+BC	Coffee husk	30	24	42	4
WL+BC	Wool	30	24	42	4
WD+BC	Wood	30	24	42	4

### Testing and Analysis

Thermogravimetric analysis (TGA) was performed in a Shimadzu TGA-60 analyzer. Samples were heated at a constant heating rate of 10 °C/min where the final temperature inside the furnace was 595 °C and the carrier gas (Argon) flow rate was 50 ml/min.

The reaction-to-fire properties of the biocomposites under radiative heat were determined using a cone calorimeter (FTT Limited, East Grinstead, UK) following ASTM E1354 standard. The external heat flux was 50 kW/m^2^ indicating a temperature of 750 °C.

Tensile moduli and strengths of the composites were measured in accordance with the ASTM D638 protocol. Three-point bending test was undertaken to measure flexural moduli and strengths of the composites based on the ASTM D790 protocol. Scanning electron microscopy (SEM) was performed using Philips XL30S Field Emission Gun scanning electron microscope, Netherlands, to study the morphology of the fracture surfaces and the residue from burning of the composites.

## Results and Discussions

### Thermogravimetric Analysis (TGA) of Individual Biomasses and Composites

**Fig. 1:**
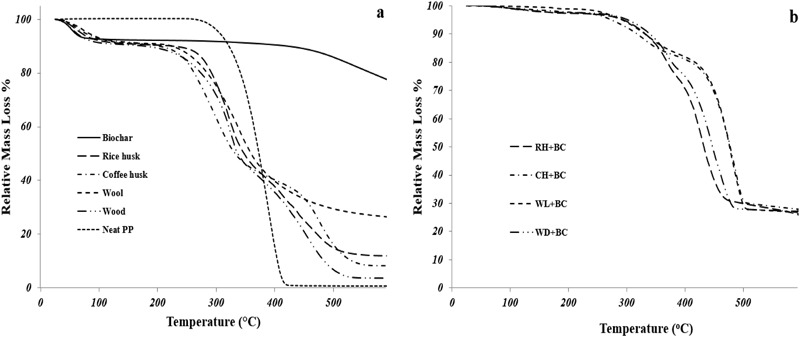
TGA analyses (**a** Mass loss curves of biomasses along with biochar and neat PP and **b** Mass loss curves of composites)

Figure 1 indicates the TGA curves of all the four biomasses compared with biochar and neat PP. Figure 1 also presents the TGA curves of the composites made from the biomasses and biochar. Table 2 also illustrates the TGA results for all the samples, such as the onset of decomposition temperature (*T*_onset_) and the temperatures at the maximum mass loss rates (T_max1_ and T_max2_ obtained from the derivatives of the mass loss curves) wherever applicable. From Fig. 1a, it can be clearly observed that biochar was the most thermally stable having negligible decomposition events and retaining the highest amount of carbonaceous residues (Das et al. 2017). Neat PP, on the other hand, was the least thermally stable as it rapidly decomposed leaving almost no residue. Amongst the four biomasses, wool was found to have the highest amount of residue which was due its charring ability (Kim et al. 2015). Rice and coffee husk retained somewhat similar amounts of residues, which was slightly higher than the residues produced by the wood. The onset of thermal decomposition of all the biomass appeared earlier compared to those of the biochar and neat PP (Table 2). The extent of decomposition was the lowest in biochar due to its thermal stability. The stable aromatic nature of carbon molecules and C-C covalent bonds amongst the molecules most probably imparted a resistance towards thermal degradation. The neat PP was seen to have a high magnitude of decomposition with a single decomposition maximum at ~389 °C. Rice husk, coffee husk, and wood all had dual decomposition maxima, one at ~300–330 °C and another one at ~440–478 °C. The first decomposition was represented by the degradation of cellulose in these biomasses while the second decomposition was marked by the degradation of lignin (Das et al. 2015a). The wool, lacking the aforementioned biopolymers, had a single decomposition maximum centered around 338 °C. The thermal decomposition in wool was dominated by the disruption of the microfibril-matrix structure along with the disulfide bonds and the peptides in the wool’s protein (Kim et al. 2017). The coffee husk had the highest *T*_max2_ (478 °C) amongst all the four biomasses. Relatively high amount of nitrogen released from the caffeine in coffee husk (Saenger et al. 2001) can suppress the volatile gases from the biomass and slow down the reaction of material, so decomposition can be delayed.

**Table 2:** TGA results individual biomasses, biochar, and their composites

Samples	*T*_onset_ (°C)	*T*_max1_ (°C)	*T*_max2_ (°C)
Neat PP	295.7	–	387.8
Biochar (BC) (TCP 900)	535.4	–	–
Rice Husk (RH)	259.5	323.6	440.4
Coffee Husk (CH)	239.4	303.6	477.8
Wool (WL)	247.6	–	337.7
Wood (WD)	199.5	332.9	452.9
RH+BC composite	270.7	369.7	413.0
CH+BC composite	308.6	323.9	489.1
WL+BC composite	297.2	–	480.6
WD+BC composite	305.5	371.5	447.1

From Fig. 1b it can be observed that all the composites had onset temperatures which were higher than the individual onset temperatures of the biomasses. This is because the thermally stable biochar in the composite delayed the start of degradation. The biomasses, when added with biochar in PP composite produced similar amounts (~27 wt%) of residues which were higher than the individual residues produced by the biomasses (although the neat wool produced a similar residue of ~26 wt%) and the neat PP (~0 wt%). This can also be attributed to the very high amount (~78 wt%) of residues left by the thermally stable biochar (Fig. 1a). All the composites had two events of decomposition maximum, one at ~330–370 °C and the other at ~412–490 °C. The first decomposition maximum was due to the degradation of cellulose in the biomasses (and breakage of microfibril-matrix structure in the wool). The decomposition maximum of neat PP has been shifted from ~389 °C to ~412–490 °C. This behavior is attributed to the inclusion of thermally stable biochar in the composite. Hence, it demonstrates that the application of biochar can induce beneficial thermal stability in composites.

### Reaction-To-Fire Properties of Individual Biomasses and Composites

**Fig. 2:**
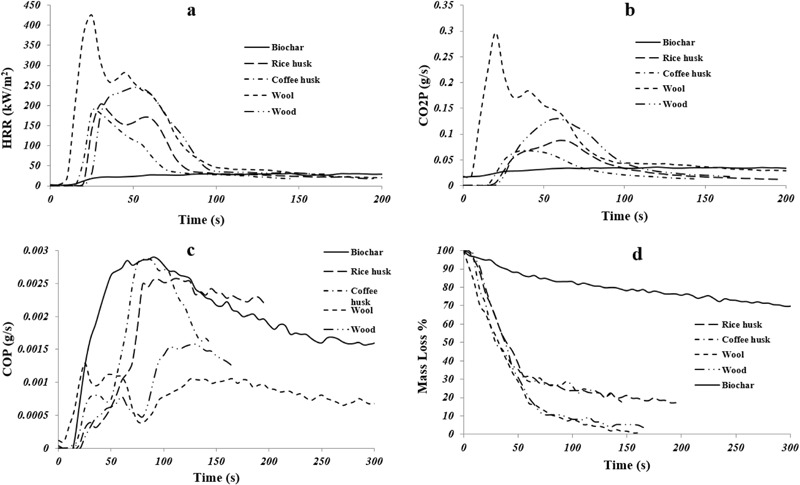
Cone calorimeter results of individual biomasses and biochar (Biochar result obtained from Das et al. 2017)

Figure 2 shows a comparison of the reaction-to-fire properties of individual biomasses and biochar as obtained from the cone calorimeter. Figures 2a, 2b, 2c and 2d represent HRR, CO_2_ production, CO production, and mass loss rate, respectively. Table 3 summarizes the other important fire properties of biomasses and biochar. The peak heat release rate (PHRR) is one of the most important parameters to judge the fire characteristics of a material whereas the HRR curve describes the overall combustion behavior of the material. The time to ignition (TTI) and time to PHRR (TPHRR) provide the time a material takes to catch fire and produce the maximum heat, respectively under radiative heat. Therefore, material ignition can be analyzed by TTI. Both the HRR and TTI depend on the exterior heat flux, degree of ventilation, and the extent of destruction of the tested material whereas the total heat release (THR) represents the internal energy of the material irrespective of the environmental factors (Schartel and Hull 2007). All the biomasses ignited rapidly (~3–8s) and also had flameout within 100 s. The wool had the highest peak heat release rate (PHRR) of 430 kW/m^2^ whereas the coffee husk had the lowest (188 kW/m^2^). The lowest T_max2_ of wool and the highest *T*_max2_ of coffee husk (amongst the biomasses) might have contributed to their respective PHRRs in cone calorimeter (Table 2). The rice husk exhibited a PHRR value of 200 kW/m^2^ whereas the wood had a PHRR of 250 kW/m^2^. The wood had the highest TTI and TPHRR amongst all the other biomasses which are positive attributes. Due to rapid combustion and early flameout, the THR of all the biomasses were fairly low (below 20 MJ/m^2^). From the HRR curves, the initial rapid combustion of wool can be clearly seen as a sharp peak at about 25 s. The disassociation of the microfibril-matrix structure and disruption of disulfide bonds triggered the char formation and once the char was built-up, the HRR went down. Under the continuous heat radiation, the char started breaking its structure and underlying wool continued decomposition and combustion, consequently resulting in another subdued heat release peak at ~45 s. However, the HRR reduced after that due to the formation of char and the extinction of fuel and continued a receding pattern until flameout at ~160 s. The rice husk also exhibited two subdued peaks and probably the second peak was a result of underlying un-burnt virgin biomass being available to the incoming O_2_ resulting in an increase in HRR. Both coffee husk and wood have a single peak implying a uniform combustion of the tested biomasses under radiative heat. The CO_2_ production of wool was the highest followed by wood, rice husk, and coffee husk (Fig. 2b). On the contrary, wool and wood had lower CO production compared to those of coffee and rice husks (Fig. 2c). From the mass loss curves (Fig. 2d), although wool was seen to leave carbonaceous residues in TGA (Fig. 1a), the continuous radiative heat caused oxidation of the chars formed, leading to complete loss of mass. The biochar left the highest amount of residues and the residues remaining in rice and coffee husk were slightly more than wood, which corroborates the TGA mass loss results observed in Fig. 1a.

**Table 3:** Reaction-to-fire properties of individual biomasses and biochar (Biochar result obtained from Das et al. 2017)

Samples	Time to ignition (TTI) (s)	Peak heat release rate (PHRR) (kW/m^2^)	Total heat release (THR) (MJ/m^2^)	Time to PHRR (TPHRR) (s)
Biochar	–	33.3 ± 4.6	9.2 ± 1.9	305.0 ± 348.0
RH	6.0 ± 1.6	200.0 ± 3.6	8.83 ± 0.4	45.0 ± 24.0
CH	3.0 ± 0.0	188.5 ± 0.9	6.05 ± 0.1	27.5 ± 4.1
WL	4.5 ± 0.7	429.8 ± 6.5	19.6 ± 1.9	25.0 ± 0.0
WD	8.5 ± 0.8	254.3 ± 16.3	11.9 ± 0.1	57.5 ± 12.0

It was observed that the biochar did not undertake ignition under the radiative heat whereas all the biomasses ignited rapidly (Das et al. 2017). Moreover, the PHRR values of the biomasses were quite high (~188–430 kW/m^2^) compared to that of the biochar (~33 kW/m^2^) and the time it took (25–57 s) to reach PHRR was also much faster than that of the biochar (~305 s). Biochar also produced the lowest amount of CO_2_ amongst all other biomasses. The somewhat inverse trend of CO_2_ and CO production can be observed in Fig. 2c. This is attributed to the greater extent of combustion (hence oxidation) of samples producing more CO_2_, and consequently, less CO. The TPHRR of biochar was difficult to measure owing to the steady state progression of its combustion cycle. The reaction-to-fire properties of the biochar are described in detail elsewhere (Das et al. 2017). In brief, owing to the high temperature of biochar production, all the flammable volatiles escaped. In addition, the high production temperature caused gradual condensation of carbon into aromatic clusters thereby making the C-C covalent bonds strong and stable. As a consequence, the tested biochar was inert under radiative heat and did not ignite.

**Fig. 3:**
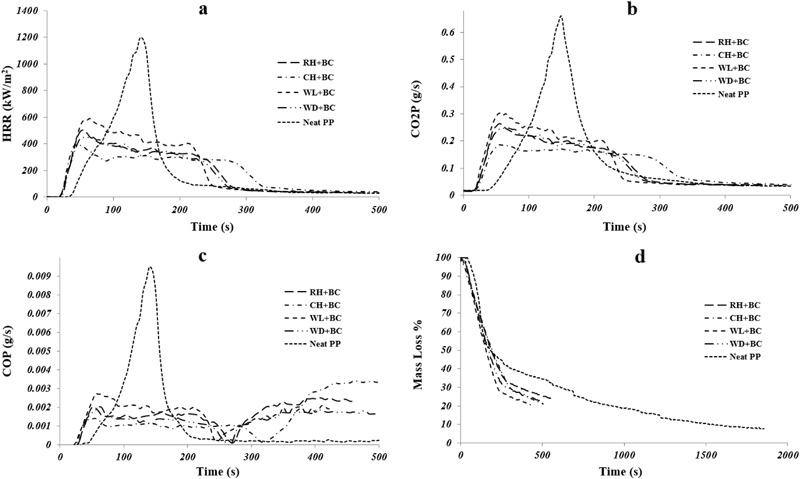
Cone calorimeter results of biomass added biochar composites

**Fig. 4:**
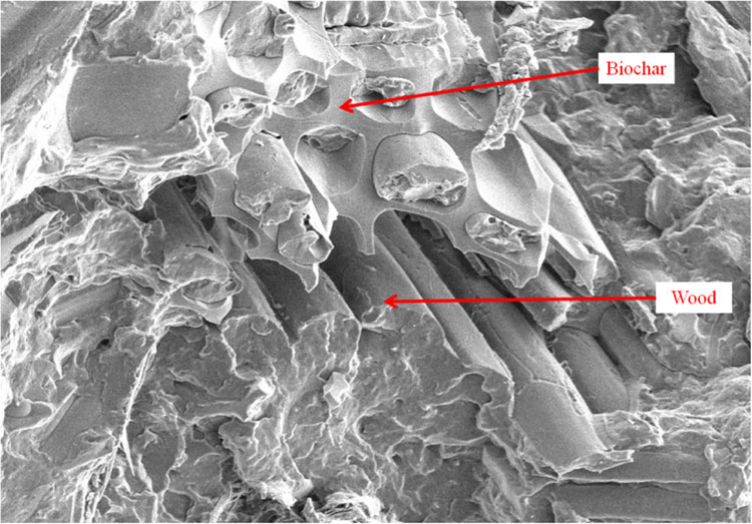
Compatibility of biochar and wood in PP matrix

Table 4 presents the cone calorimeter parameters for biomass added biochar composites whereas Fig. 3 shows the reaction-to-fire properties of the same composites. The cone calorimeter results of the composites are compared with that of the neat PP. From Table 4 and Fig. 3a, it can be observed that neat PP burned rapidly with a sharp HRR peak due to the fast degradation of its hydrocarbon backbone. The PHRR of the wool based composite was the highest (596 kW/m^2^), and the PHRR of coffee husk added composite was the lowest (394 kW/m^2^), amongst all the biomasses. The high PHRR of neat wool and low PHRR of neat coffee husk (Table 3) might have contributed towards the respective PHRRs in the composites. On the other hand, the neat rice husk, although, having lower PHRR than neat wood, its composite exhibited higher PHRR (501 kW/m^2^) than the wood-based composite (449 kW/m^2^). The possible explanation for this behavior is explained as follows. The biochar was made from the same wood, which is used as one of the four biomasses. Production of biochar retains the structure of the parent feedstock (in this case, wood) (Sun et al. 2012). Therefore, it is possible that the biochar and wood were physically in close contact with each other in the PP matrix (like pieces in a puzzle) (Fig. 4). The rice husk with a different morphology with the biochar in the PP matrix, combusted with a greater severity (compared to wood-based composite) without the shielding effect of biochar and consequently increased the overall PHRR of the composite. The prolonged combustion of wool (due to char formation) resulted in a THR similar to that of the neat PP (97 MJ/m^2^) whereas wood, rice, and coffee husk had similar THR of ~90 MJ/m^2^ (Table 4). All the biomass-based composites experienced shorter TPHRR and TTI than those of the neat PP, most probably due to an early onset of thermal decomposition and rapid ignition of the biomasses (compared to neat PP) as observed in Tables 2 and 3. The CO_2_ production curves (Fig. 3b) of the biomass-based biochar composites mirrored the HRR curves because CO_2_ was produced as long as the composites were burning. The peak rate of CO_2_ production was similar to the values of PHRR corresponding to each of the biomass in the composites. Hence, wool based composite had the highest rate of CO_2_ production whereas coffee husk had the lowest. Wood and rice husk based composite had more or less similar rate of CO_2_ production. The CO production increased beyond the discontinuance of CO_2_ production in all the biomass-based composites. This can be attributed to the biochar in the composite providing an insulating carbonaceous layer which facilitated an incomplete combustion of polymer to produce less CO_2_ and more CO. It is interesting to note that the HRR and gas production of the biomasses and biochar-based composites were significantly lower than those of the neat PP. Moreover, the severity of mass loss of the biomass/biochar composites is much less than that of the neat PP. Therefore, the addition of thermally stable biochar in biomass added composites is beneficial when reaction-to-fire properties are concerned. This is evident as wood added composite (without biochar) was previously reported to have a PHRR of 568 kW/m^2^ (Zhang et al. 2012) and the addition of biochar reduced the PHRR to ~21 % in the present study.

**Table 4:** Reaction-to-fire properties of biomass added biochar composites

Samples	Time to ignition (TTI) (s)	Peak heat release rate (PHRR) (kW/m^2^)	Total heat release (THR) (MJ/m^2^)	Time to PHRR (TPHRR) (s)
Neat PP	29.0 ± 2.0	1054.0 ± 120.0	97.0 ± 14.0	120.0 ± 18.0
RH+BC	17.0 ± 0.94	501.8 ± 15.9	90.8 ± 3.3	51.6 ± 2.7
CH+BC	16.3 ± 0.54	394.1 ± 3.5	90.8 ± 1.0	45.0 ± 0.0
WL+BC	16.3 ± 1.97	596.4 ± 29.2	97.6 ± 0.1	60.0 ± 4.7
WD+BC	17.3 ± 1.45	449.1 ± 3.2	87.5 ± 1.7	58.3 ± 7.2

### Mechanical Properties of the Composites

The tensile (tensile strengths and moduli) and flexural properties (flexural strengths and moduli) of all the composites and neat PP are summarized in Table 5. Among the different biomass added composites, the wood-based sample had the highest tensile/flexural strength values (36 MPa/67 MPa) and flexural modulus (4.4 GPa). Its tensile modulus (4.73 GPa) was close second to that of the rice husk based composite (4.87 GPa). The tensile strength of rice husk composite was similar to that of the neat PP (~32 MPa) but its flexural strength was higher (62.6 MPa). In fact, the flexural strength and tensile/flexural moduli of all the biomass-based composites were higher than that of the neat PP. The good dispersion of the biochar in the PP matrix and the biochar pore infiltration by PP enhanced the stress transfer efficiency between the matrix and the constituents. In addition, the inclusion of biochar with the biomasses reduced the deformability of the PP which consequently enhanced the flexural strengths and tensile/flexural moduli. The tensile properties of the composites can depend on the individual chemical composition of the biomasses (Fidelis et al. 2013). The cellulose is the main component responsible for the resistance of the biomass towards stress. This strong and stiff constituent of biomass (i.e. cellulose) remains aligned with longitudinal axis of the fibers which contribute to the fiber’s mechanical properties. The average amount of cellulose in the lignocellulosic biomasses is as follows: pine wood 40–45% (Fengel and Grosser 1975); rice husk 25–35 % (Luduena et al. 2011); coffee husk 19–26 % (Bekalo and Reinhardt 2010). Therefore, the wood-based composite had the highest resistance (consequently highest tensile strength) due to the highest amount of cellulose in it. Similarly, the higher amount of cellulose in rice husk compared to coffee husk resulted in better mechanical properties. In this research, short wool fibers (average length: 2.8 mm) were used to manufacture the composites. This length was lower than the critical length of the wool (3.6 mm), which is essential for maximizing the strength properties of the resulting composites (Kim et al. 2014).

**Table 5:** Mechanical properties of the biomass added biochar composites compared with neat PP

Samples	Tensile strength (MPa)	Tensile modulus (GPa)	Flexural strength (MPa)	Flexural modulus (GPa)
Neat PP	32.6 ± 0.2	1.5 ± 0.0	51.0 ± 0.0	1.6 ± 0.0
RH+BC	32.7 ± 0.3	4.8 ± 0.1	62.6 ± 0.9	4.1 ± 0.0
CH+BC	29.2 ± 0.2	4.2 ± 0.1	57.9 ± 0.8	4.3 ± 0.0
WL+BC	29.9 ± 0.8	3.5 ± 0.0	57.0 ± 0.6	3.3 ± 0.0
WD+BC	35.8 ± 0.1	4.7 ± 0.0	67.2 ± 0.2	4.4 ± 0.0

The addition of coffee husk and wool did not enhance the tensile and flexural strengths of the composite. However, it improved the modulus (both tensile and flexural). This was due to the reduction of intra-particle distances as a consequence of the inclusion of the higher amount of reinforcing constituents (Ho et al. 2015). Application of rice husk to biochar/PP composite had a positive correlation with flexural strength and tensile/flexural moduli. Out of all the biomasses, wood seemed to have an overall favorable effect on all the tested mechanical properties of biochar/PP composites. In past investigations where only the biomass was used (loading amount of 30 wt%- similar to current study) along with PP in the composites generally obtained inferior mechanical properties compared to what is observed in the present study. Stark and Rowlands (2003) prepared wood/PP composites and they reported the tensile/flexural strengths and tensile/flexural moduli to be 24.3 MPa/41.4 MPa and 3.46 GPa/2.89 GPa, respectively. In another study by Rodríguez et al. (2003), coffee husk/PP composites were reported to have a very low tensile strength and modulus of 12.4 MPa and 1.94 GPa, respectively. Rice husk/PP composites exhibited a tensile strength of ~ = <25 MPa (Premalal et al. 2002; Yang et al. 2004) whereas wool/PP composites had tensile strength and modulus of 34 MPa and 2.4 GPa (Kim and Bhattacharyya 2016), respectively. In the current investigation, the average tensile/flexural strengths and tensile/flexural moduli for all the biomass and biochar-based composites (combined) were 31.9 MPa/61.7 MPa and 4.3 GPa/4.05 GPa, respectively. Therefore, it can be observed that except for wool, all other biomasses, when used to make PP composites without biochar possess inferior mechanical properties. These observations point out the fact that biochar is indeed beneficial for enhancing the mechanical properties of a biomass-based composite. In addition, biochar plays an additional role in resisting flame propagation upon composite combustion.

### Microstructural Analyses of Tensile Fractured Surfaces and Combustion Chars of Composites

**Fig. 5:**
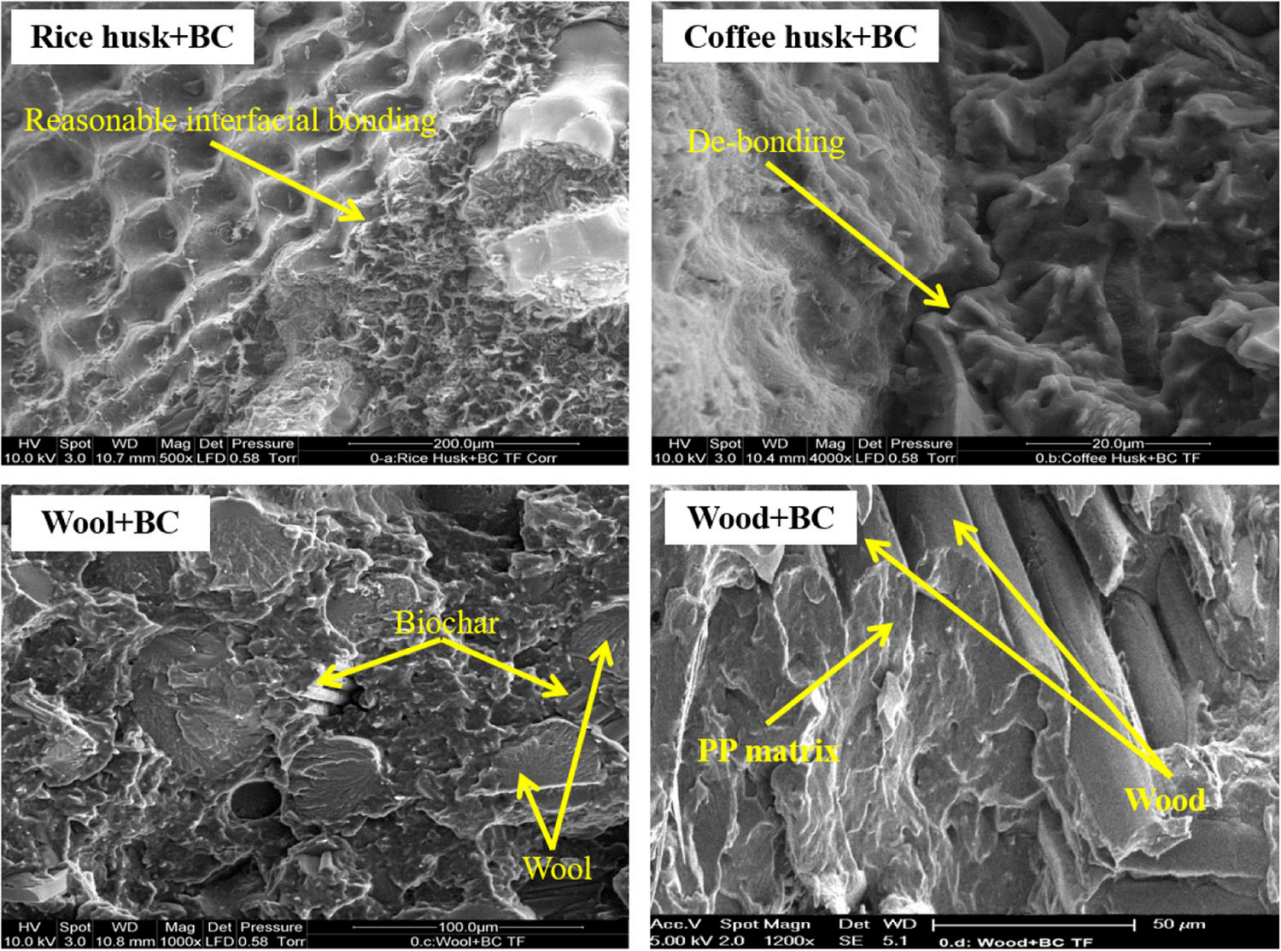
SEM images of biomass added biochar composites

**Fig. 6:**
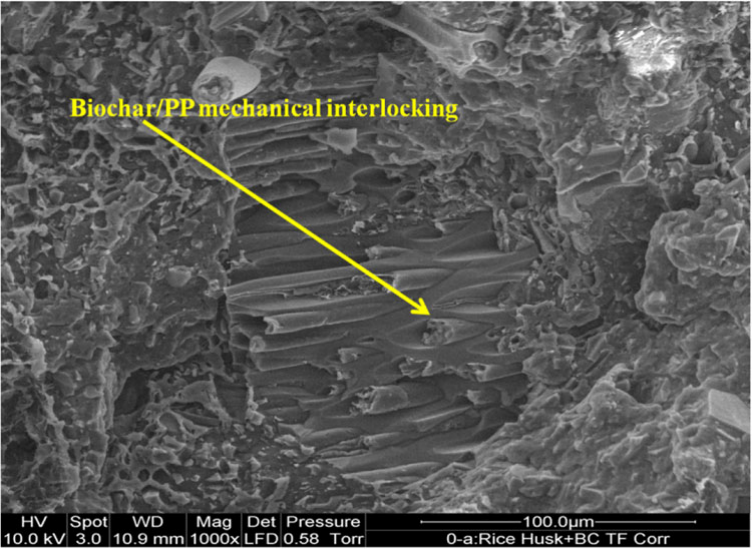
SEM image of mechanical interlocking of biochar and PP

Scanning electron microscopy (SEM) was performed to gain an insight into the state of manifestation of the biomasses in the PP matrix. From Fig. 5, it can be observed that the rice husk had reasonable interfacial bonding with the PP aided by 4 wt% MAPP, whereas the bonding of wood and PP was good (the wood+ BC image). The bonding between wood and PP is also demonstrated in Figure S1 in supplementary information. The SEM image (Figure S1) is from a different sample set that contained ammonium polyphosphate, nevertheless, the image supports the argument of bonding between wood and PP. It can be observed from Figure S1, that part of the wood has been fractured off under tensile stress whereas the remaining wood particle is still adhering to the PP matrix. On the other hand, a clear de-bonding from the PP matrix was seen with coffee husk at several places in the sample (an example is in the coffee husk+ BC image). In the wool added composite, the biochar particles were seen to be embedded within the wool fibers potentially hindering the interaction of wool and PP. Hence, the tensile strength of the wood composite was the highest followed by rice husk and then coffee husk and wool (Table 5). The mechanical interlocking of biochar with PP occurred in all the biomass added composites, exemplified here with the rice husk added sample (Fig. 6). This interlocking is the reason for the enhanced tensile/flexural moduli of the composites compared to the neat PP (Table 5).

**Fig. 7:**
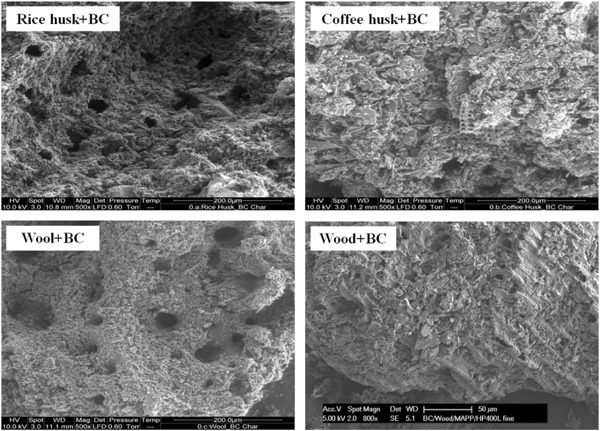
Char microstructure of biomass added biochar composites

The structural integrities of the chars obtained after combustion in the cone calorimeter were also analyzed through SEM. The SEM images of combustion chars of the biomass added composites are illustrated in Fig. 7. The chars of coffee husk and wood added with biochar seemed to be more compact and dense whereas the chars of wool and rice husk were observed to have more holes and fissures. These holes allowed the transport of O_2_ between the ambient environment and the underlying materials. This might have been the reason for coffee husk and wood-based composites to have better reaction-to-fire properties (especially PHRR) than wool and rice husk based composites (Table 4).

### Environmental and Economic Merits of the Biomass-Based Biochar Composites

The use of wood fibres, as the adequate biomass for biochar composites performance, also leads to improved environmental sustainability. It is known that wood-based thermoplastic polymer composites can be recycled at the end of their lives. Bourmaud and Baley (2007) have studied the effect of recycling on mechanical properties of composites based on cellulose fibers and PP, and identified that tensile modulus of the composite is preserved after reprocessing cycles. Moreover, biochar is also known as a sustainable material, thus the hybrid of wood and biochar in the composites leads towards sustainability and environmental innocuousness (Das et al. 2017). Furthermore, the natural fibers emit much less toxic gases, such as CO and NO_x_, consume less energy and have more disposal options compared to those of synthetic materials, namely glass and carbon fibers, during their manufacturing and incineration. Therefore, the bio-based composites have shown better performance in life cycle assessment than synthetic fiber based composites (Joshi et al. 2004).

As mentioned in Introduction section, the biomasses used in the current study are organic wastes. Employment of the wastes and biochar in composites manufacturing can significantly reduce total production cost, as the price of biochar (NZD 0.22/kg) is much less than natural and synthetic fibers. The economic advantages of the materials can also enhance potential of the hybrid of biomass and biochar for mechanically sound and fire resistant composites.

## Conclusions

In this paper, four biomass wastes (rice husk, coffee husk, course wool, and landfilled wood) were added with biochar and PP to manufacture composites. The composites were analyzed for their fire and thermal properties using cone calorimetry and thermogravimetry. Their tensile and flexural properties were also measured accordingly using Instron Universal Testing Machine. The individual reaction-to-fire analysis of the neat biomasses revealed that the coffee husk had the lowest PHRR value. However, upon fabrication of biochar-based composites with these biomasses, the wood-based composite, was found to have the best mechanical properties (tensile/flexural strength and moduli) and acceptable reaction-to-fire characteristics (second lowest PHRR and the lowest THR) amongst the biomasses. Therefore, the wood was identified as a suitable biomass/co-reinforcement to be used along with biochar for future manufacturing and development of biochar-based composites. However, further research is warranted where biochar prepared from rice husk, coffee husk, and wool should be employed to make composites. It is recommended to observe if the parent biomass is compatible with their respective biochars in the composite through the results of reaction-to-fire and mechanical tests.

## Electronic supplementary material

Supplementary Information
