# Supplementary material for: An Attempt to Find a Suitable Biomass for Biochar-Based Polypropylene Biocomposites
Source: Environ Manage. 2018 Mar 28;62(2):403–13. doi: 10.1007/s00267-018-1033-6 (PMC6060818; doi:10.1007/s00267-018-1033-6)
Supplement: Supplementary file 1 — Supplementary Information [file 267_2018_1033_MOESM1_ESM.docx]

**An attempt to find a suitable biomass for biochar based polypropylene biocomposites**

^1*^Oisik Das, ^2^Nam Kyeun Kim, ^1^Mikael S Hedenqvist, ^2^Richard J.T. Lin, ^3^Ajit K Sarmah, ^2^Debes Bhattacharyya

*^1^Department of Fibre and Polymer Technology- Polymeric Materials, School of Chemical Sciences and Engineering, KTH Royal Institute of Technology, Stockholm 100 44, Sweden*

*^2^Centre for Advanced Composite Materials, Department of Mechanical Engineering, University of Auckland, New Zealand*

*^3^Department of Civil and Environmental Engineering, Centre for Advanced Composite Materials, University of Auckland, New Zealand*

**Supplementary Information**

**^*^Corresponding author:**

Oisik Das

Department of Fibre and Polymer Technology

Polymeric Materials Division

School of Chemical Science and Engineering, KTH Royal Institute of Technology,

Teknikringen 58 Stockholm 100 44, Sweden

Tel: +46 790 469 886. E-mail: [oisik@kth.se](mailto:oisik@kth.se)


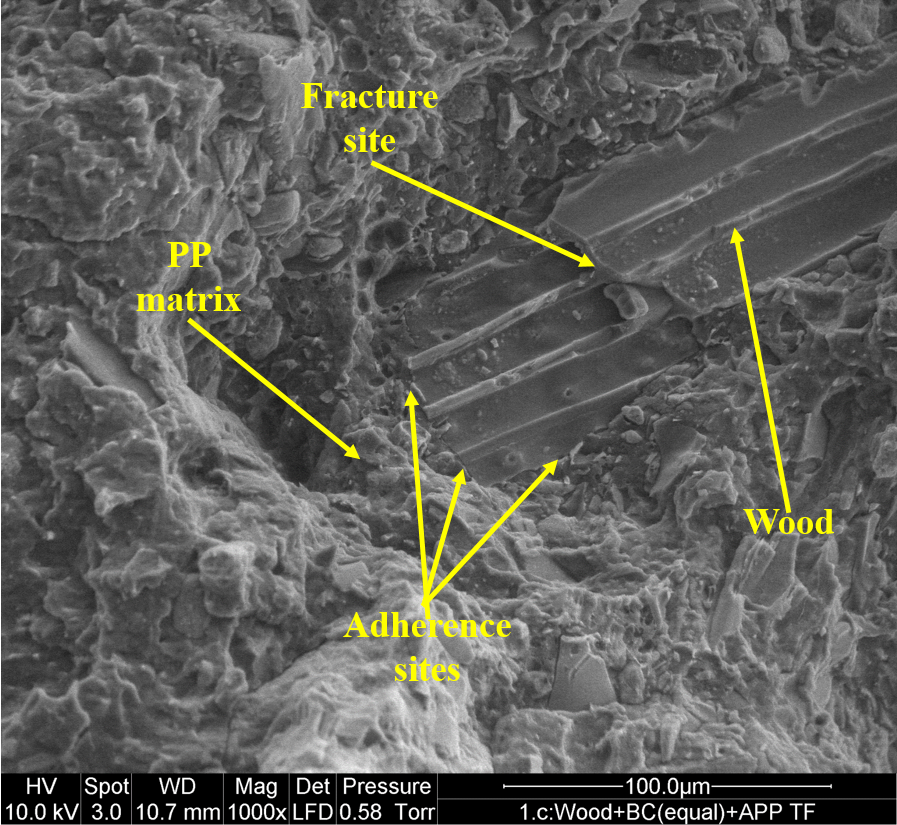


**Figure S1:** The wood fractured under tensile stress whereas the remaining wood is still adhering to PP matrix through a good interfacial bonding.
